# Comparative genomics approach to build a genome-wide database of high-quality, informative microsatellite markers: application on *Phytophthora sojae*, a soybean pathogen

**DOI:** 10.1038/s41598-019-44411-z

**Published:** 2019-05-28

**Authors:** Guohong Cai, Tomara J. Fleury, Ning Zhang

**Affiliations:** 1 Crop Production and Pest Control Research Unit, USDA-ARS, 47907 West Lafayette, IN USA; 2 Botany and Plant Pathology Department, Purdue University, 47907 West Lafayette, IN USA; 3 Department of Plant Biology, Rutgers, The State University of New Jersey, 08901 New Brunswick, NJ USA; 4 Department of Biochemistry and Microbiology, Rutgers, The State University of New Jersey, 08901 New Brunswick, NJ USA

**Keywords:** Genomic analysis, Pathogens

## Abstract

Microsatellites are a tract of repetitive, short DNA motifs (usually 1 to 6 bp) abundant in eukaryotic genomes. They are robust molecular markers in many areas of studies. Development of microsatellite markers usually involves three steps: (1) obtaining microsatellite-containing sequences, (2) primer design, and (3) screening microsatellite loci for polymorphism. The first and third steps require considerable resources. Next generation sequencing technologies have greatly alleviated the constraint of the first step. In this study, we leveraged the availability of genome assemblies of multiple individuals in many species and designed a comparative genomics approach to bioinformatically identify polymorphic loci. Our approach can eliminate or greatly reduce the need of experimental screening for polymorphism and ensure that the flanking regions do not have length difference that would confound interpretation of genotyping results using microsatellite markers. We applied this approach on *Phytophthora sojae*, a soybean pathogen, and identified 157 high-quality, informative microsatellite markers in this oomycete. Experimental validation of 20 loci supported bioinformatics predictions. Our approach can be readily applied to other organisms of which the genomes of multiple individuals have been sequenced.

## Introduction

Microsatellites, also known as simple sequence repeats (SSRs) or short tandem repeats (STRs), are a tract of repetitive, short DNA motifs (usually 1 to 6 bp). Microsatellites are valuable molecular markers. They are abundant in an organism’s genome, often occur at thousands of locations and cover most of the genome. Microsatellites have higher mutation rate than other areas of the genome^1,2^, resulting in higher genetic diversity. Slippage during DNA replication, in concert with unequal cross-over, is generally accepted as the main mechanism of microsatellite mutation^3^. This mode of mutation can result in multiple alleles at a locus, which are inherited co-dominantly. Microsatellite loci with higher repeat numbers tend to have higher mutation rate^1^. These attributes make microsatellites a robust tool in many research areas, such as molecular-assisted breeding, population genetics, genealogy and genome mapping^4^.

Genotyping using microsatellite markers usually involves PCR amplification of a microsatellite locus using primers from its flanking regions. The sizes of PCR amplicons can be resolved by gel electrophoresis or more accurately, by capillary electrophoresis implemented in a commercial genetic analyzer^5,6^. A microsatellite locus is considered to be informative if it has multiple alleles within a species or a population under study. Length difference between PCR amplicons is expected to reflect different repeat numbers of the microsatellite motif^5,7–9^.

Traditionally, the development of microsatellite markers follows these steps: (1) genomic DNA is fragmented, microsatellite-containing fragments can be enriched (usually through affinity capture), and the enriched microsatellite-containing DNA fragments are cloned and sequenced; (2) primer pairs are designed from the flanking regions; and (3) the primer pairs are screened using a small subset of individuals to identify polymorphic microsatellite loci — those having multiple alleles in the tested individuals. Steps 1 and 3 above require considerable time and resources. The advent of next-generation sequencing and its low cost revolutionized the process. Abundant genome and transcriptome sequences are now available for many species, or can be easily obtained with low cost from yet-to-be sequenced species. Microsatellite loci can be routinely identified from raw sequence reads or assembled genomes obtained through next-generation sequencing^8–11^, thus alleviating the constraint of step 1. However, the identified microsatellite loci still need to be screened through PCR to determine which ones are polymorphic. Usually, a large number of loci need to be screened to obtain sufficient number of polymorphic loci. For example, in *Anisogramma anomala*, a fungal pathogen of hazelnut, 23 loci were found to be polymorphic among 236 loci screened^9^; in *Fusarium virguliforme*, a fungal pathogen on soybean, 12 polymorphic loci were identified in a screening of 92 microsatellite loci with very high repeat numbers^8^; and in *Phytophthora sojae*, an oomycete pathogen on soybean, 21 out of 158 microsatellite loci with extremely high repeat numbers (minimal 15 repeats) were found to be polymorphic among 33 isolates^12^. Additionally, it is usually assumed that the flanking regions of microsatellite loci are conserved and the length difference in PCR amplicons is due to varying repeat numbers of the microsatellite motifs. This assumption may not always be true^9^ and can confound downstream analysis and lead to inaccurate conclusions.

With the advance of genomics, multiple individuals of representative genetic background have been sequenced in many species. This allows identification of polymorphic microsatellite loci directly from these genome assemblies, alleviating the constraint of step 3 mentioned above. It also allows us to ensure that the selected loci have conserved flanking regions so that the length difference in PCR amplicons indeed reflects varying repeat numbers of the microsatellite motif. In this study, we define a microsatellite locus as high-quality and informative if it has multiple alleles in a species, and its flanking regions are conserved and will not interfere with deducing repeat numbers of the target microsatellite motif from the length of PCR amplicons. We combined the power of a microsatellite motif identification software program, an *in vitro* PCR software program and our own post-processing pipeline to identify genome-wide, high quality, informative microsatellite loci by mining multiple genomes within a species, and we applied this approach on *Phytophthora sojae*.

*Phytophthora sojae* is an oomycete that causes root and stem rot in soybean, one of the most destructive soybean diseases in North America^13^. This species has a diploid genome, except during the brief gamete phase. It is homothallic (i.e., self-mating), though occasional outcrossing was evidenced^14–16^. Thus, limited difference between the two copies of genomic DNA is expected.

## Results and Discussion

### Bioinformatics pipeline

**Figure 1:**
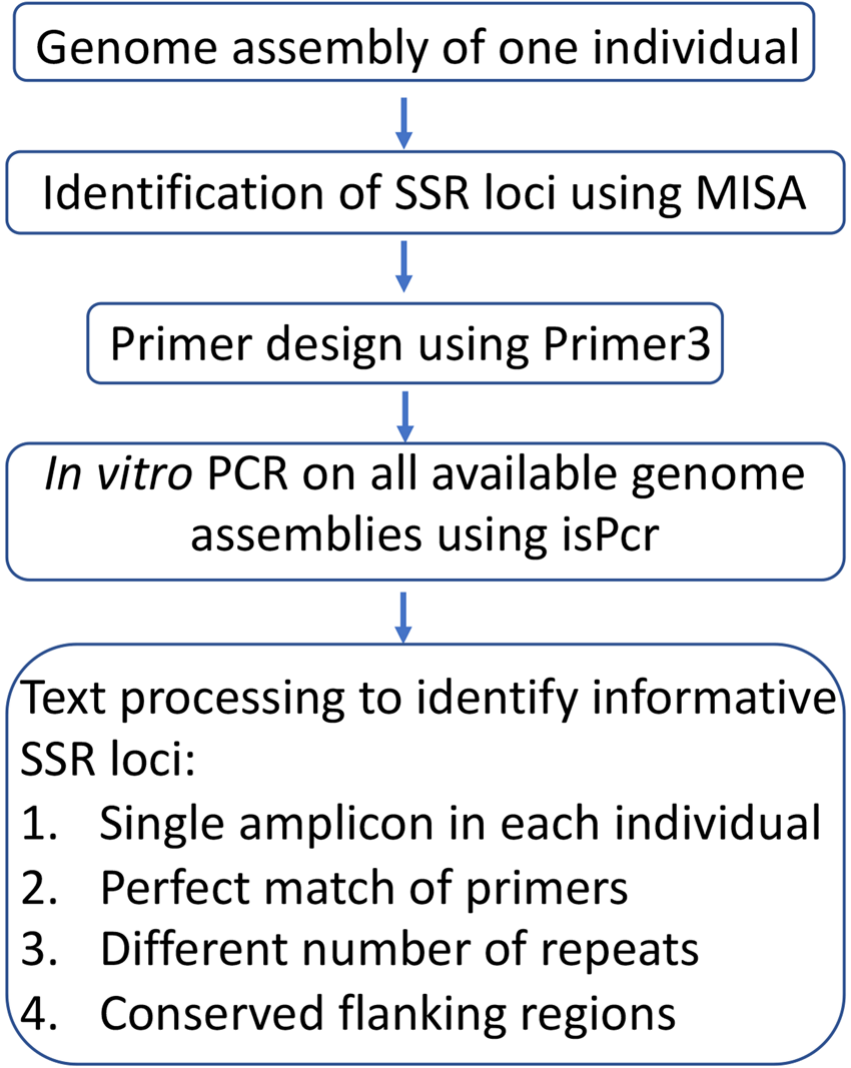
Bioinformatics pipeline for identifying high-quality, informative microsatellite loci using comparative genomics approach.

Our approach is illustrated in the flowchart in Fig. 1. Starting with one genome assembly, usually the one that is most complete and of best quality, microsatellite loci with mono- to hexa-nucleotide motifs are identified using the MISA program^17^. Primer3 program^18^ is then used to design primer pairs for di- to hexa-nucleotide motifs. Loci with mono-nucleotide motifs are excluded because of the difficulty in accurately differentiating alleles with 1 bp length difference. Microsatellite loci with compound motifs (two motifs immediately next to each other) are also excluded because, when these loci are used in genotyping and without sequencing the amplicons, it would be difficult or impossible to determine which motif causes length difference between alleles, and two alleles could have identical length but different sequences (e.g., allele *a* has two more repeats of a bi-nucleotide motif while allele *b* has one more repeat of the adjacent tetra-nucleotide motif).

**Figure 2:**
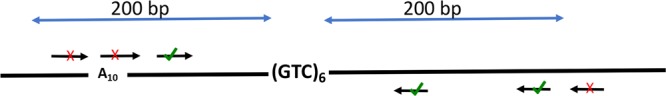
Flanking regions allowed for primer design.

Primer pairs are designed in the 200 bp flanking regions on both sides of each target locus. However, when there is another microsatellite locus nearby (including those with mono-nucleotide motifs and compound loci), primers are required to locate in-between the loci so that PCR amplicon will not include more than one microsatellite locus (Fig. 2).

*In Silico* PCR is performed using the isPcr program (Jim Kent, University of California, Santa Cruz) to simulate PCR reactions in all individuals in this species whose genomes have been sequenced and publicly available. The results are then processed using custom Perl scripts to identify high-quality, informative loci and their associated primer pairs based on the following requirements: (1) the primer pair only produces a single amplicon in each individual; (2) the termini of the amplicon match the primer sequences perfectly; (3) there are different number of repeats of the microsatellite motif among the individuals; and (4) for loci with di- or tri-nucleotide motifs, there is no length difference of the flanking sequences, and for loci with tetra- to hexa-nucleotide motifs, there is at most 1 bp length difference in the flanking regions.

### Application on *P*. *sojae*

The genomic sequences of four strains of *P*. *sojae*, P6497, P7064, P7074 and P7076, were analyzed. Though *P*. *sojae* is diploid, their genomes are assembled into haploid form, just as many non-monoploid genomes currently available. These strains belong to races 2, 7, 17 and 19, respectively (https://chassintranet.ucr.edu/phyto/). P7064 was isolated from Ontario, Canada, while the other three strains were isolated from Mississippi, USA. P6497 was sequenced using Sanger chemistry^19^, and the other three strains were re-sequenced using 454 technology.

Microsatellite loci were first identified in the genome assembly of P6497, which captured much higher portion of the genome and was more continuous. A total of 12,098 microsatellite loci, including 126 in compound formation, were identified across the 82 scaffolds. The density of microsatellite loci was 146/Mb (45/Mb excluding those with mono-nucleotide motifs). Other than loci with mono-nucleotide motifs (69.2%), loci with tri-nucleotide motifs (17.9%) were most abundant, followed by those with di-, tetra-, penta- and hexa-nucleotide motifs (9.9%, 2.0%, 0.5% and 0.5%, respectively). Other than mono-nucleotide motifs, AGC/CTG motif (and all its iterations) was most frequent (780 loci), followed by AG/CT (503 loci) and AC/GT (496 loci).

Primers were designed for the 3,473 loci with di- to hexa-nucleotide motifs that were not in compound formation and there were enough flanking sequences (minimal 21 bp) on both sides to allow primer design. With our criteria, at least one primer pair was found for 3,447 loci, while five primer pairs were picked for 3,445 loci. In total, 17,233 primer pairs were picked for downstream analysis. After *in silico* PCR using genome assemblies of all four strains as templates, the results were processed to identify high-quality, informative microsatellite loci according to the criteria we described above. In total, we identified 678 primer pairs for 157 loci (PsSSR1-PsSSR157) in which there were various number of repeats and flanking regions that met our requirements (Supplementary Table S1). We also identified 176 polymorphic loci whose flanking regions had length difference exceeding our selection limit.

Of the 157 loci that met our requirements, 52 loci had primer pairs that were predicted to amplify from all four strains, while the other 105 only had primer pairs that would amplify from two or three strains (Supplementary Table S1). *P*. *sojae* has an estimated 1n genome size of 95 Mb while the assemblies range from 50.1 to 82.6 Mb. Large portions of the genome are missing in these assemblies and three of the four assemblies are highly fragmented. Therefore, many of the 105 loci likely exist in all four strains, but the expected amplicons are either missing in one or more genome assemblies, or not assembled into the same contig.

### Experimental verification

Twenty loci of the 157 loci identified above were experimentally validated through PCR. Thirteen of these 20 loci had predicted amplicons from all four strains based on *in silico* PCR, while the other seven loci only had predicted amplicons from two or three strains (Table 1). DNA fragments of expected sizes were successfully amplified from all four strains for the 20 loci (Table 1). For those with expected amplicons by *in silico* PCR, experimental validation perfectly matched the predictions. For those without expected amplicons, experimental PCR produced products with sizes that allowed unambiguous deduction of the number of repeats. This confirmed that the failure of *in silico* PCR in those cases was due to incompleteness and/or fragmentation of the genome assemblies.

**Table 1:** Experimental validation of microsatellite loci identified through comparative genomics analysis.

SSR locus	Motif	SSR location on P6497 assembly	Forward primer	Reverse primer	# of repeats^a^			
					P6497	P7064	P7074	P7076
PsSSR1	GT	NW_009258115.1:496894 + 496905	GGCGGCTCCGTAGATTCTAG	TACAGATCTTGCCAGCACCG	6/6	6/6	5/5	6/6
PsSSR5	AAGAA	NW_009258115.1:2742556 + 2742585	TGGAGAAGGACAACAAGGCG	TGAATCTAGCGGCTGTGTCC	6/6	7/7	6/6	NA/6
PsSSR9	GCT	NW_009258115.1:6849281 + 6849301	CCGCAGTATTCTGAGCACGA	GTCCAACTGCTGCGACTACT	7/7	7/7	6/6	7/7
PsSSR13	AT	NW_009258115.1:9137994 + 9138005	GCCCTTGGGGTTTGTGTCTA	AAGCCTTAGCCCAGAGAGGA	6/6	6/6	7/7	6/6
PsSSR17	GT	NW_009258115.1:11175261 + 11175284	ATCGACAGAGCAAACGCAGA	CCACGTACGAGCGCACTATT	12/12	12/12	10/10	12/12
PsSSR18	TGC	NW_009258115.1:11380520 + 11380549	TGCAGTAGGGGCATGTGTTG	CGACGGTACCTCACACCAAA	10/10	10/10	8/8	8/8
PsSSR30	AGCC	NW_009258116.1:5307746 + 5307769	GCGAGCCATTCCCAGTTCTA	TCGAGGGCGATACATCATGC	6/6	4/4	4/4	NA/6
PsSSR33	ACA	NW_009258116.1:5982108 + 5982140	TTCCATCCCACGTTCTTCGG	AGCCACAACATCACTGCGTA	11/11	NA/10	14/14	11/11
PsSSR35	AGT	NW_009258116.1:6503023 + 6503067	TGTGTATGCCCTCGAGCTTG	GAGCATCACGTGTTCGCATC	15/15	8/8	8/8	8/8
PsSSR41	TCG	NW_009258116.1:9732910 + 9732930	TACAGCTGACTCGGTCGAGA	ACGGACGAAATCGACGACTC	7/7	7/7	8/8	8/8
PsSSR44	TC	NW_009258116.1:10645448 + 10645481	TGGGGATACATTGCATTCTCGT	CGTGCAGTGAAAAGAGTGGC	17/17	NA/17	12/12	NA/17
PsSSR47	GCACCT	NW_009258117.1:351303 + 351344	CTGTGGACGACGTTGCTACT	CGCACAAGAGAAGCAGGAAC	7/7	6/6	7/7	NA/6
PsSSR50	CTA	NW_009258117.1:722986 + 723015	CCGTCATGTCCACGAACAGA	GAGAACCAAACACAGCGCTG	10/10	10/10	11/11	10/10
PsSSR56	TATT	NW_009258117.1:2466499 + 2466522	CGACGCCGATTCAATTGCTT	CCTCGTCAGCAGCTACAACA	6/6	6/6	8/8	6/6
PsSSR58	ACA	NW_009258117.1:4215171 + 4215200	CGTGATGTCCTGGCCCATAA	GAAGGAGAAGGAGATCCGCG	10/10	13/13	13/13	10/10
PsSSR69	ATGCG	NW_009258117.1:8979503 + 8979532	TGCTCGACAACTCTCTGCAG	CGGAGAAATCAGGTCGCCAT	6/6	6/6	8/8	NA/6
PsSSR76	GTC	NW_009258118.1:1806329 + 1806346	AGTTGCAGCCTCTGAAGCTT	GAGTCTGCCACAGATCCCAC	6/6	5/5	5/5	5/5
PsSSR79	CAG	NW_009258118.1:2645205 + 2645225	AGTGACCGACAACAGCTCAG	TTCTTGACCGAGCTGCTGAG	7/7	6/6	7/7	7/7
PsSSR86	TTGCAC	NW_009258118.1:6470526 + 6470555	AGGATCTCCTCGTCCACCTC	TGCACTGGAAGAAAACCGGT	5/5	4/4	4/4	5/5
PsSSR97	GCT	NW_009258119.1:4196084 + 4196113	CTTGAAGCGGAGGAGGTCAC	GGACAGCAACGGTTTCTTCG	10/10	9/9	7/7	NA/10

^a^Numbers before the slash are based on genome mining and *in silico* PCR; and those after the slash are based on experimental validation. NA, not available.

### Comparing with a previous study

In a previous study, Dorrance and Grunwald screened 158 microsatellite loci in *P*. *sojae* with at least 15 repeats^12^. These loci were identified by screening cDNA sequences of *P*. *sojae*^20^. They found 21 loci to be polymorphic. We looked for those loci in our database. Excluding the three compound loci (PS20, PS24 and PS25) that were not analyzed in our study, only three of the remaining 18 loci were included in our database (PS16 = PsSSR65, PS29 = PsSSR35 and PS38 = PsSSR115 in Supplementary Table S1). To address the discrepancy, we performed *in silico* PCR using their primers^12^. Eleven loci (PS01, PS05, PS06, PS10, PS12, PS17, PS18, PS19, PS27, PS33, and PS36) had predicted PCR product only from one strain, P6497; and one locus (PS04) did not have predicted PCR product in any strain. BLASTN searches of the *P*. *sojae* genome assemblies using their reported amplicon sequences as query showed that these sequences either were missing from these genome assemblies, or broken, usually at or near the microsatellite sequences.

For the remaining three loci, PS07 was eliminated in our analysis because it had two predicted amplicons in strain P6497. These two amplicons were located on the same contig (NW_009258117.1), indicating that the two amplicons were paralogs, not homologs - alleles of the same locus in a diploid genome. PS30 had predicted amplicons from two strains, P6497 and P7076. It had 18 repeats of the motif (AGTC) in P6497, but a point mutation at the third repeat turned it into an imperfect microsatellite locus and led it to be erroneously characterized as 15 repeats in the previous study^12^. PS37 had predicted products from strains P6497 and P7076, and they both had 16 repeats of the motif (GAG). However, in strain P7076, there was an 1 bp insertion after repeat 6 and could lead to incorrect binning in downstream analysis. Use of these loci could potentially lead to incorrect conclusion.

### Considerations of comparative genomics approach

Here we describe a comparative genomic approach to identify high-quality, informative microsatellite loci genome-wide. Two benefits of our approach are apparent: (1) It alleviates the burden of step 3 in microsatellite marker development: screening loci for polymorphism. Instead of screening a large number of loci to obtain a sufficient number of polymorphic loci^8,9,12^, our approach requires no or minimal experimental screening. (2) It is often assumed that microsatellite loci have conserved flanking regions, and that length difference in PCR amplicons is due to varying repeat numbers of the target loci. However, our analysis showed that this assumption is not necessarily valid - 176 out of 333 polymorphic loci did not meet our requirement due to length difference of their flanking regions. Our approach ensures that only loci with flanking regions conserved in length are selected. The accuracy of this approach depends on the accuracy of genome assembly.

The key requirement of our approach, of course, is the availability of genome assemblies from multiple individuals in a species. This requirement is being met by more and more species, and it is not difficult or resource-consuming to obtain genome sequences from multiple individuals for the organisms for which no or only one genome assembly is available.

Another consideration is the genetic background of these individuals whose genomes have been sequenced. If these individuals are of similar genetic background while the whole species has much larger genetic diversity, many loci that are identical in the sequenced individuals may in fact be polymorphic in the species. On the other hand, if the sequenced individuals cover much of species diversity, when you use the identified loci to study a population with narrow genetic background, many identified polymorphic loci may be identical in that particular population.

Completeness and continuity of the genome assemblies have a large impact on our approach. Missing sequences and/or broken assemblies will lead to polymorphic loci not being identified. This was the reason that 12 out of 18 loci in a previous study^12^ were not included in our database. Moreover, of the 3,447 loci we analyzed, many loci had predicted amplicons in one to three strains and they were not found to be polymorphic. It’s reasonable to expect some of those loci may be polymorphic, but are not identified due to incompleteness and/or fragmentation of the genome assemblies. Missing regions in genome assemblies tend to be enriched in repetitive sequences. Polymorphic microsatellite loci in repetitive regions will be challenging when used in genotyping, due to difficulty in designing primers that will specifically amplify the target loci.

Accuracy of genome assemblies can also affect our analysis. Incorrect assemblies can mislead every step in the analysis, from microsatellite loci identification, primer design, to *in silico* PCR. In this study, we paid particular emphasis on mono-polymers (microsatellite loci with mono-nucleotide motifs). Mono-polymer region is prone to mis-assembly (e.g., those based on 454 sequencing technology). We set a low threshold for identifying mono-nucleotide motifs and ensured that the PCR amplicons would not contain such sequences. The good agreement between experimental validation and bioinformatics analysis in our study showed that this is not a big concern for *P*. *sojae* (Table 1).

With more and more individuals being sequenced in many species, and the genome assemblies becoming more complete, more continuous and more accurate, our approach can be applied in many more organisms and significantly simplify the process of microsatellite marker development. Our approach assumes haploid genomes, or diploid and polyploid genomes assembled in haploid form. This is the current status for most organisms. Polymorphisms within an individual are lost in these genome assemblies. However, our approach can be readily applied if each ploidy of a diploid or polyploid genome is separately assembled. For example, if the diploid *P*. *sojae* genome is teased apart into two haploid assemblies, each ploidy can be treated as a separate assembly in our analysis.

## Materials and Methods

### Data sources

The genome assembly of strain P6497 was downloaded from GenBank (RefSeq assembly accession no.: GCF_000149755.1) with an assembly size of 82.6 Mb. The assemblies of strains P7064, P7074 and P7076 were downloaded from eumicrobedb.org with assembly sizes ranged from 50.1 to 51.8 Mb. In addition to being more complete, the genome assembly of P6497 is also more continuous: it has contig N50 of 386 kb, while the other assemblies have contig N50 of 3 kb or less.

### Data analysis

Microsatellite loci were identified using software package MISA^17^ with the following parameters: for mononucleotide motifs, minimal 8 repeats; dinucleotide motifs, minimal 6 repeats; tri- to hexa- nucleotide motifs, minimal 5 repeats; and minimal distance between neighboring loci for identification of compound loci, 0 bp. Up to five primer pairs were designed for all loci with di- to hexa- nucleotide motifs using Primer3^18^ with the following requirements: product size 100–250 bp, primer length 18–25 bp (optimal 20 bp), primer melting temperature 57–63 °C (optimal 60 °C), primer GC ratio 30–70% (optimal 50%). All primers were required to be located within 200 bp flanking regions on each side of but with a minimal 3 bp away from the target loci. If there was another microsatellite locus (including those with mono-nucleotide motifs) within the 200 bp flanking regions of a target locus, the amplicon was required not to overlap the neighboring locus. *In silico* PCR was conducted using the software package isPcr version 3.3 (http://genome-test.cse.ucsc.edu/∼kent/) with default parameters. Custom Perl scripts were used for text processing.

### Experimental validation

*Phytophthora sojae* strains P6497, P7064, P7074 and P7076 were obtained from the World *Phytophthora* Genetic Resource Collection (https://chassintranet.ucr.edu/phyto) and maintained on half-strength lima bean agar. For DNA extraction, the strains were grown in half-strength lima bean broth at room temperature for 7 days. DNA was extracted from harvested mycelium using the Plant DNeasy Mini kit (Qiagen).

Nested PCR reactions were performed with three primers: forward primer with a M13 sequence (TGTAAAACGACGGCCAGT) appended to its 5′ end, reverse primer (Table 1), and M13 primer labeled with 5′-FAM. PCR reactions were performed in duplicates in 15 μl containing 1x Amplitaq Gold Buffer (Applied Biosystems), 2 mM MgCl_2_, 0.2 mM mixed dNTPs, 0.6 U Amplitaq Gold DNA Polymerase (Applied Biosystems), 40 nM forward primer, 160 nM reverse primer, 160 nM 5′FAM-labeled M13 primer, and 10 ng template DNA. An initial denaturing cycle at 95 °C for 5 min was followed by 38 cycles of denaturing at 95 °C for 30 s, annealing for 45 s at 58 °C for the first 25 cycles and 54 °C for the remaining 13 cycles, and extension at 72 °C for 45 s, and a final extension cycle at 72 °C for 15 min. The sizes of PCR amplicons were resolved by capillary electrophoresis on an ABI 3730XL Genetic Analyzer together with GeneScan™ 600 LIZ™ dye Size Standard v2.0 (Applied Biosystems). Allele sizes were determined with the aid of software Peak Scanner version 2.0 (Applied Biosystems). Raw genotyping data is provided in Supplementary Table S2.

### Disclaimer

Mention of trade names or commercial products in this publication is solely for the purpose of providing specific information and does not imply recommendation or endorsement by the U.S. Department of Agriculture. USDA is an equal opportunity provider and employer.

## Supplementary information

Table S1

Table S2

## Data Availability

All data generated or analyzed during this study are included in this published article (and its Supplementary Information files).
